# Duration of adenosine-induced myocardial hyperaemia: insights from quantitative 13N-ammonia positron emission tomography myocardial perfusion imaging

**DOI:** 10.1093/ehjci/jeae096

**Published:** 2024-04-08

**Authors:** Chrysoula Garefa, Dominik F Sager, Pascal S Heiniger, Susanne Markendorf, Tobia Albertini, Stjepan Jurisic, Marko Gajic, Catherine Gebhard, Dominik C Benz, Aju P Pazhenkottil, Andreas A Giannopoulos, Philipp A Kaufmann, Piotr J Slomka, Ronny R Buechel

**Affiliations:** Department of Nuclear Medicine, Cardiac Imaging, University and University Hospital Zurich, NUK A 12, Ramistrasse 100, Zurich 8091, Switzerland; Department of Nuclear Medicine, Cardiac Imaging, University and University Hospital Zurich, NUK A 12, Ramistrasse 100, Zurich 8091, Switzerland; Department of Nuclear Medicine, Cardiac Imaging, University and University Hospital Zurich, NUK A 12, Ramistrasse 100, Zurich 8091, Switzerland; Department of Nuclear Medicine, Cardiac Imaging, University and University Hospital Zurich, NUK A 12, Ramistrasse 100, Zurich 8091, Switzerland; Department of Nuclear Medicine, Cardiac Imaging, University and University Hospital Zurich, NUK A 12, Ramistrasse 100, Zurich 8091, Switzerland; Department of Nuclear Medicine, Cardiac Imaging, University and University Hospital Zurich, NUK A 12, Ramistrasse 100, Zurich 8091, Switzerland; Department of Nuclear Medicine, Cardiac Imaging, University and University Hospital Zurich, NUK A 12, Ramistrasse 100, Zurich 8091, Switzerland; Department of Nuclear Medicine, Cardiac Imaging, University and University Hospital Zurich, NUK A 12, Ramistrasse 100, Zurich 8091, Switzerland; Department of Nuclear Medicine, Cardiac Imaging, University and University Hospital Zurich, NUK A 12, Ramistrasse 100, Zurich 8091, Switzerland; Department of Nuclear Medicine, Cardiac Imaging, University and University Hospital Zurich, NUK A 12, Ramistrasse 100, Zurich 8091, Switzerland; Department of Nuclear Medicine, Cardiac Imaging, University and University Hospital Zurich, NUK A 12, Ramistrasse 100, Zurich 8091, Switzerland; Department of Nuclear Medicine, Cardiac Imaging, University and University Hospital Zurich, NUK A 12, Ramistrasse 100, Zurich 8091, Switzerland; Division of Artificial Intelligence in Medicine, Imaging, and Biomedical Sciences, Department of Medicine, Cedars-Sinai Medical Center, Los Angeles, CA, USA; Department of Nuclear Medicine, Cardiac Imaging, University and University Hospital Zurich, NUK A 12, Ramistrasse 100, Zurich 8091, Switzerland

**Keywords:** myocardial perfusion imaging, adenosine, duration, hyperaemic effect, artefact

## Abstract

**Aims:**

This study aimed to assess the impact of adenosine on quantitative myocardial blood flow (MBF) in a rapid stress–rest protocol compared with a rest–stress protocol using 13N-ammonia positron emission tomography (PET) myocardial perfusion imaging (MPI) and to gain insights into the time dependency of such effects.

**Methods and results:**

Quantitative MBF at rest (rMBF) and during adenosine-induced stress (sMBF) and myocardial flow reserve (MFR) were obtained from 331 retrospectively identified patients who underwent 13N-ammonia PET MPI for suspected chronic coronary syndrome and who all exhibited no perfusion defects. Of these, 146 (44.1%) underwent a rapid stress–rest protocol with a time interval (Δ*t*_stress–rest_) of 20 ± 4 min between adenosine infusion offset and rest imaging, as per clinical routine. The remaining 185 (55.9%) patients underwent a rest–stress protocol and served as the reference. Groups did not differ regarding demographics, risk factors, medication, left ventricular function, and calcium scores. rMBF was significantly higher in the stress–rest vs. the rest–stress group [0.80 (interquartile range 0.66–1.00) vs. 0.70 (0.58–0.83) mL·min^−1^·g^−1^, *P* < 0.001], and, as sMBF was identical between groups [2.52 (2.20–2.96) vs. 2.50 (1.96–3.11), *P* = 0.347], MFR was significantly lower in the stress–rest group [3.07 (2.43–3.88) vs. 3.50 (2.63–4.10), *P* = 0.007]. There was a weak correlation between Δ*t*_stress–rest_ and rMBF (*r* = −0.259, *P* = 0.002) and between Δ*t*_stress–rest_ and MFR (*r* = 0.163, *P* = 0.049), and the proportion of patients with abnormally high rMBF was significantly decreasing with increasing Δ*t*_stress–rest_.

**Conclusion:**

Intravenously applied adenosine induces a long-lasting hyperaemic effect on the myocardium. Consequently, rapid stress–rest protocols could lead to an overestimation of rMBF and an underestimation of MFR.

## Graphical Abstract

**Graphical Abstract:**
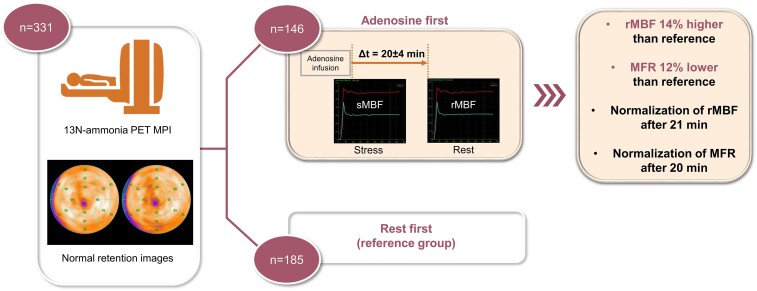
PET MPI, positron emission tomography myocardial perfusion imaging; sMBF, stress myocardial blood flow; rMBF, rest myocardial blood flow; MFR, myocardial flow reserve.

## Introduction

Functional imaging is pivotal in the diagnosis and risk stratification of patients with known or suspected chronic coronary syndrome (CCS), and pharmacological stress agents such as adenosine and regadenoson play a common role among imaging modalities for the assessment of ischaemia, particularly in nuclear cardiology and cardiovascular magnetic resonance (CMR). Results from a longitudinal survey in Germany show that 47% of patients undergoing single-photon emission computed tomography (SPECT) myocardial perfusion imaging (MPI) unable to exercise were stressed with adenosine.^1^ Similarly, due to its favourable safety profile and tolerability, adenosine is the most frequently used stress agent in CMR.^2,3^

Regadenoson has a terminal elimination half-life of around 2 h.^4^ Therefore, if regadenoson is applied in a stress-first protocol, aminophylline, a non-selective phosphodiesterase inhibitor, is commonly administered post-stress, presumably resulting in a complete return of coronary artery hyperaemia to a pre-stress level.^5^ Contrary, however, and despite the scarcity of the literature on the duration of adenosine-induced hyperaemia, mainly focusing on invasive coronary blood flow velocity measurements during and after systemic adenosine application,^6–8^ post-stress imaging after application of adenosine is commonly considered feasible without the need for any antagonists assuming that the hyperaemic effects of adenosine are expected to be entirely resolved after 10–15 min.^5^

Current US guidelines for nuclear MPI merely state that rest imaging is typically followed by stress imaging. However, stress-first is considered feasible with quantitative positron emission tomography (PET) MPI.^9^ In contrast, the current European guidelines call for caution concerning stress–rest protocols as ‘the residual effects of the pharmaceutical stress agent on perfusion could affect the resting images’.^10^ Finally, current recommendations for stress CMR simply mandate a waiting time of at least 10 min between stress and rest imaging.^11^

The current study aimed to assess the impact of adenosine on quantitative myocardial blood flow (MBF) measurements in a rapid stress–rest protocol compared with a rest–stress protocol using 13N-ammonia PET MPI. Furthermore, we aimed to gain insights into the time dependency of such effects.

## Methods

### Patient population

This is a retrospective single-centre study with patients who underwent 13N-ammonia PET MPI scans for exclusion of CCS between January 2017 and March 2023. From the hospital’s electronic health records database, we identified and screened all patients who exhibited no perfusion defects (i.e. normal stress and rest retention images) as per routine clinical assessment and underwent pharmacologically induced vasodilation with adenosine. The local ethics committee approved the study (BASEC-Nr. 2023-01220), and all patients provided written informed consent.

### PET acquisition, reconstruction, and analysis

All patients underwent clinically indicated PET MPI using 13N-ammonia acquired at rest and during pharmacological stress with adenosine infused at 0.14 mg·kg^−1^·min^−1^ over 6 min. Importantly, and as per institutional routine, rest imaging was randomly acquired either before (rest–stress) or after (stress–rest) stress imaging. The rationale behind this routine was to optimize resource utilization by using one 13N-ammonia production for two simultaneous MPI scans on two PET scanners located side by side. Hence, patients underwent a stress–rest or rest–stress protocol with one physician/cardiologist sequentially stressing one patient and then the other. Importantly, no particular choice was made regarding which patient underwent stress or rest imaging first. For stress imaging, after 3 min into adenosine infusion, a body mass index (BMI)-adapted dose of 13N-ammonia was injected into an antecubital vein. Data were acquired in list mode on a PET scanner (Discovery MI PET/CT, GE Healthcare, Waukesha, WI, USA) as previously reported.^12^ The data sets were reconstructed using ordered subset expectation maximization (OSEM, VUE Point HD, 2 iterations, 16 subsets), and a 5 mm Hanning filter and standard decay, scatter, and sensitivity corrections (voxel size 2.34 mm) were applied. Unenhanced computed tomography (CT) was used for attenuation correction of PET/CT data sets. Regardless of the imaging sequence, 13N-ammonia activity for the second acquisition was two-fold that of the first dose, resulting in a 5:1 dosing differential at the time of the second scan compared with the residual activity still present from the first. The time interval between the beginning of the stress and rest PET data acquisition was recorded from DICOM headers, and the time interval between the offset of adenosine infusion and the beginning of the rest PET data acquisition (Δ*t*_stress–rest_) was then calculated by deducting 180 s of the time difference. The quantitative MBF measurements obtained from the rest–stress imaging sequence served as the reference and internal validation standard. Additional unenhanced electrocardiogram (ECG)-triggered CT scans were acquired for the calculation of the coronary artery calcium (CAC) score where clinically indicated. PET image acquisition was acquired in list mode over a total of 14 min. For both stress and rest, dynamic data sets were reconstructed from the first 7 min of acquisition and consisted of nine frames of 10 s duration, six frames of 15 s, three frames of 20 s, two frames of 30 s, and one frame of 120 s. Static and ECG-gated data sets were reconstructed from the last 10 min of the acquisition. MBF during stress (sMBF) and at rest (rMBF; corrected for the rate–pressure product and for residual activity, as previously described^13^), myocardial flow reserve (MFR), and the left ventricular ejection fraction (LVEF) at rest were calculated using commercially available software (QPET 2017.7, Cedars-Sinai Medical Center, Los Angeles, CA, USA) as well as a research version of QPET for residual activity correction.

### Statistical analysis

Statistical analysis was performed using SPSS (version 29.0, IBM Corporation, Armonk, NY, USA) and MedCalc (version 19.6.4, MedCalc Software Ltd, Ostend, Belgium). Normally distributed continuous variables are expressed as mean ± standard deviation. Otherwise, the median and interquartile range (IQR; 25^th^–75th percentile) are given. Spearman correlation analysis was applied for non-normally distributed variables. Categorical variables are represented as absolute numbers and percentages. Unpaired *t*-tests were used for the comparison of normally distributed continuous variables. The χ^2^ test was used for the comparison of dichotomous variables. Mann–Whitney *U* und Kruskal–Wallis tests were applied for non-normally distributed variables. Multiple linear regression analysis was used to determine significant predictors of rMBF and MFR, and standardized coefficients (β) are provided. The Benjamini–Hochberg procedure was used to control the false discovery rate at 10%. Using the rest–stress group as a reference, we calculated the upper and lower limits of normal for rMBF, sMBF, and MFR. Therefore, the 5th percentile for the left-sided reference interval (i.e. for rMBF) and 95th percentile for the right-sided reference intervals (i.e. for sMBF and MFR) were calculated after testing for outliers and following the Clinical and Laboratory Standards Institute (CLSI) guidelines. All statistical tests were two-tailed, and a *P*-value of <0.05 was considered statistically significant.

## Results

We identified a total of 334 patients eligible for this study. Data analysis and/or residual activity correction failed in three patients due to corrupted PET data sets. Of the remaining 331 patients, 146 (44.1%) underwent a stress–rest protocol, while the remainder underwent a rest–stress protocol. The time interval between both scans in the stress–rest group was 20 ± 4 min. Total protocol duration (including image acquisitions and the time interval between) amounted to 55.4 ± 34.5 min for the rest–stress group and 47.9 ± 31.5 min for the stress–rest group. Injected 13N-ammonia activity was 331 ± 127 and 308 ± 121 MBq for stress and rest, respectively. A CAC score was available in 213 (64.4%) patients.

**Figure 1:**
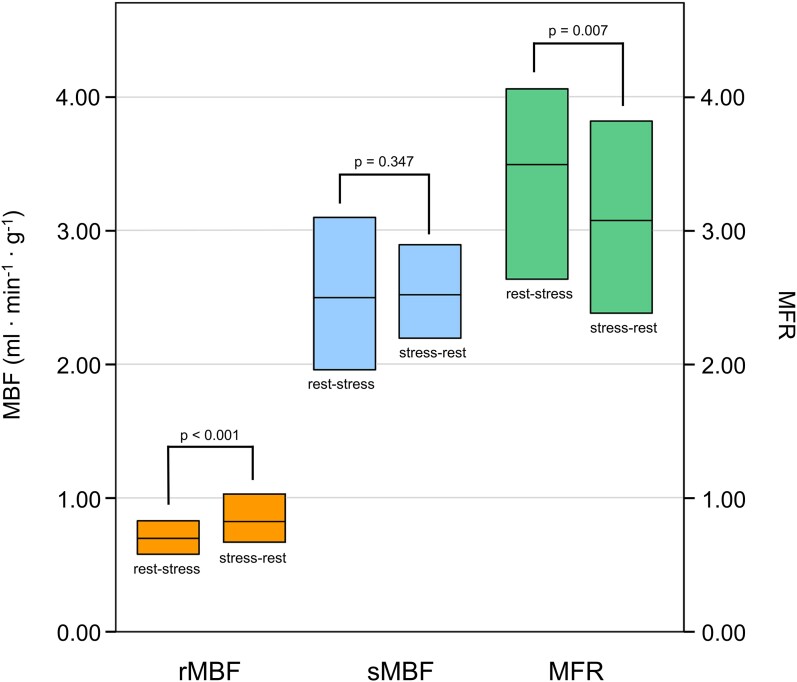
Boxplots of rMBF, sMBF, and MFR values of patients in the rest–stress vs. stress–rest group. Boxes comprise the IQR and horizontal bars depict the median.

*Table 1* exhibits the demographics and characteristics of the overall population stratified according to the imaging protocols. Demographics, cardiovascular risk factors, and cardiac medication did not differ significantly between patients who underwent a stress–rest vs. rest–stress protocol. In contrast, we found that rMBF and, as sMBF remained comparable, MFR differed significantly between groups, resulting in a 14% higher rMBF and a 12% lower MFR in patients undergoing a stress–rest vs. those undergoing a rest–stress protocol (*Figure 1*). rMBF and MFR values without correction for the rate–pressure product are provided in Supplementary data online, *Table S1*.

**Table 1:** Patient demographics, clinical and imaging characteristics

		Imaging sequence		
	All patients (*n* = 331)	Stress–rest (*n* = 146)	Rest–stress (*n* = 185)	*P*-value
Age (years)	63 ± 12	64 ± 10	62 ± 14	0.172
Male sex	209 (63.1%)	86 (58.9)	124 (67.0)	0.156
Body mass index (kg·m^−2^)	28.3 ± 6.6	28.4 ± 6.2	28.2 ± 7.0	0.410
Haemodynamics at rest imaging				
Heart rate (b.p.m.)	66 (60–77)	66 (59–74)	68 (60–79)	0.182
Systolic blood pressure (mmHg)	136 (123–149)	140 (127–159)	134 (120–146)	**0.008**
Rate–pressure product (mmHg·b.p.m.)	9306 (7550–10 971)	9414 (7900–10 817)	9165 (7463–11 220)	0.649
Cardiovascular risk factors				
Hypertension	145 (43.8)	71 (48.6)	74 (40.0)	0.116
Dyslipidaemia	79 (23.9)	41 (28.1)	38 (20.5)	0.110
Diabetes	66 (19.9)	30 (20.6)	36 (19.5)	0.806
Positive family history	33 (10.0)	17 (11.6)	16 (8.6)	0.366
Smoking	66 (19.9)	36 (24.7)	30 (16.2)	0.056
Cardiac medication				
Antithrombotics	63 (19.0)	34 (23.3)	29 (15.7)	0.091
Beta-blockers	65 (19.6)	32 (21.9)	33 (17.8)	0.354
Angiotensin-converting enzyme inhibitor (ACEI)/angiotensin receptor blocker (ARB)	90 (27.2)	38 (26.0)	51 (27.6)	0.673
Lipid-lowering drugs	70 (21.2)	38 (26.0)	32 (17.3)	0.059
Imaging findings				
LVEF (%)	63 ± 10	63 ± 9	64 ± 10	0.671
CAC score^a^	68 (8–415)	81 (9–321)	44 (2–444)	0.308
rMBF (mL·min^−1^·g^−1^)	0.75 (0.61–0.95)	0.80 (0.66–1.00)	0.70 (0.58–0.83)	**<0**.**001**
sMBF (mL·min^−1^·g^−1^)	2.52 (2.07–3.02)	2.52 (2.20–2.96)	2.50 (1.96–3.11)	0.347
MFR	3.22 (2.60–4.01)	3.07 (2.43–3.88)	3.50 (2.63–4.10)	**0**.**007**

Significant *P*-values are indicated in bold letters.

Values given are mean ± SD, and absolute numbers and percentages are in parentheses or median and IQR in brackets.

^a^Available in 213 patients (107 in the stress–rest and 106 in the rest–stress group).

While heart rates did not differ statistically significantly between groups, the median systolic blood pressure was higher than in the reference group. However, the rate–pressure products did not differ significantly. Importantly, there was no significant correlation between rMBF and heart rate (*P* = 0.466), systolic blood pressure (*P* = 0.353), or the rate–pressure product (*P* = 0.295) in the stress-first group.

Multiple regression analysis was run to predict rMBF from imaging sequence, sex, age, BMI, LVEF, and all cardiovascular risk factors. The model statistically significantly predicted rMBF [*F*(10 320) = 10.679, adjusted *r*^2^ = 0.227, *P* < 0.001]. Among all variables, imaging sequence (β = 0.257, *P* < 0.001), sex (β = −0.193, *P* < 0.001), BMI (β = −0.337, *P* < 0.001), and LVEF (β = 0.170, *P* < 0.001) added statistically significantly to the prediction. Similarly, for MFR, multiple regression analysis including the same variables revealed that the model weakly but statistically significantly predicted MFR [*F*(10 320) = 4.692, adjusted *r*^2^ = 0.101, *P* < 0.001]. Among all variables, imaging sequence (β = −0.156, *P* = 0.006) and hypertension (β = −0.156, *P* = 0.006) added statistically significantly to the prediction.

In a subanalysis confined to the subpopulation where CAC scores were available (*n* = 213), multiple regression analysis identified the same independent predictors for rMBF as given above [*F*(11 201) = 7.332, adjusted *r*^2^ = 0.247, *P* < 0.001], and, of note, the CAC score *per se* was not found to be an independent predictor (*P* = 0.303). In contrast, the same model failed to predict MFR [*F*(11 201) = 2.204, adjusted *r*^2^ = 0.059, *P* = 0.016].

### Time dependency of adenosine effects on MBF

**Figure 2:**
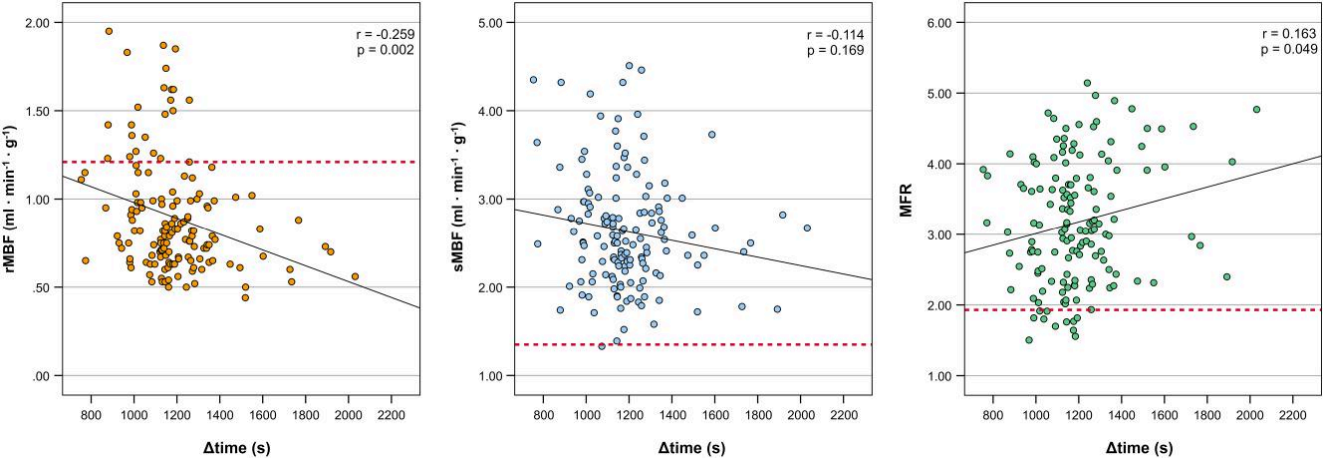
Scatterplots for rMBF, sMBF, and MFR values as derived from 13N-ammonia PET of patients within the stress–rest group in relation to Δ*t*_stress–rest_. Dotted lines denote the upper limit of normal for rMBF (at 1.21 mL·min^−1^·g^−1^) and lower limits of normal for sMBF and MFR (at 1.34 and 1.93 mL·min^−1^·g^−1^, respectively).

In an analysis confined to the stress–rest group, we found a weak but statistically significant correlation between Δ*t*_stress–rest_ and rMBF (*r* = −0.259, *P* = 0.002) and between Δ*t*_stress–rest_ and MFR (*r* = 0.163, *P* = 0.049) but not between Δ*t*_stress–rest_ and sMBF (*r* = −0.114, *P* = 0.169) (*Figure 2*). Of note, there was no significant correlation between Δ*t*_stress–rest_ and heart rate (*P* = 0.765), systolic blood pressure (*P* = 0.100), or rate–pressure product (*P* = 0.178).

After the identification of the upper and lower limits of normal for rMBF (i.e. >1.21 mL·min^−1^·g^−1^), sMBF (i.e. <1.34 mL·min^−1^·g^−1^), and MFR (i.e. <1.93), we assessed for both groups the proportion of patients, which exhibited MFR and rMBF values outside these normal limits. Overall, the proportion of patients with an rMBF above the normal proportions was 4.3% (8/185) vs. 15.1% (22/146) in the rest–stress vs. the stress–rest group, respectively (*P* < 0.001). In contrast, the proportion of patients with an sMBF below normal was 3.8% (7/185) in the rest–stress group vs. 0.7% (1/146) in the stress–rest group (*P* = 0.082). This resulted in a proportion of patients with an MFR below normal of 4.9% (9/185) in the rest–stress group vs. 7.5% (11/146) in the stress–rest group (*P* = 0.357).

**Figure 3:**
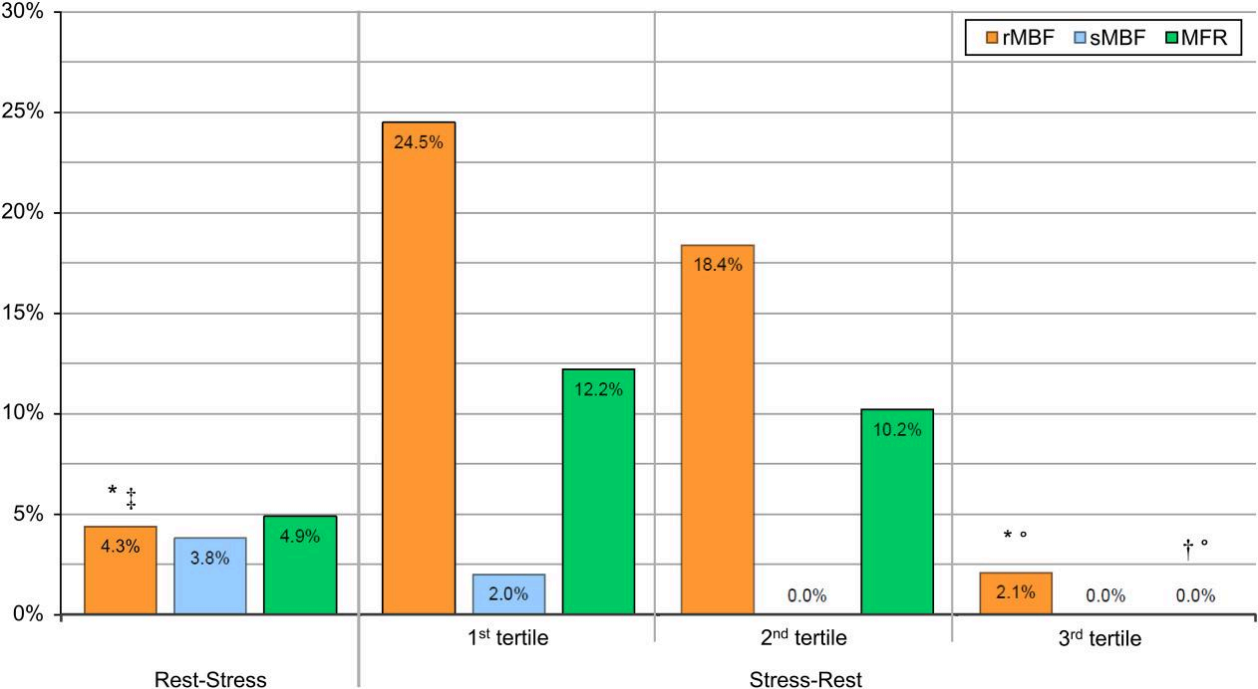
Proportion of patients with abnormal quantitative PET measurements between groups and stratified by tertiles of Δ*t*_stress–rest_. Proportion * differs significantly from the first tertile (*P* < 0.005); † differs significantly from the first tertile (*P* < 0.05); ‡ differs significantly from the second tertile (*P* < 0.005); ° differs significantly from the second tertile (*P* < 0.05).

*Figure 3* depicts the proportion of patients with abnormal quantitative PET measurements with those patients in the stress–rest group stratified by tertiles of Δ*t*_stress–rest_. The proportion of patients with abnormal rMBF differed among tertiles, and pairwise comparison revealed that the proportion of patients with the longest Δ*t*_stress–rest_ (i.e. 2.1% in tertile 3) as well as of those in the rest–stress group (i.e. 4.3%) was significantly lower compared with patients with shorter Δ*t*_stress–rest_ (i.e. 24.5% in tertile 1 and 18.4% in tertile 2). The proportion of patients with abnormal MFR also differed among tertiles in that the proportion of patients with the longest Δ*t*_stress–rest_ (i.e. 0% in tertile 3) was significantly lower compared with patients with shorter Δ*t*_stress–rest_ (i.e. 12.2% in tertile 1 and 10.2% in tertile 2). The proportions of patients with abnormal sMBF did not differ among groups. Of note, no patients exhibited rMBF values above normal after Δ*t*_stress–rest_ of 1258 s (21.0 min), and no patients showed an MFR below normal after Δ*t*_stress–rest_ of 1193 s (19.9 min).

## Discussion

In the current study, we demonstrate that MBF, as obtained from quantitative 13N-ammonia PET MPI, remains altered for a substantial duration after the offset of intravenous adenosine infusion, remaining increased by 14% after 20 min as compared with MBF of a matched cohort without prior adenosine infusion, thereby decreasing MFR by 12%. Furthermore, our results reveal insights into the temporal profile of adenosine-induced myocardial hyperaemia, hinting at a long-lasting residual effect of adenosine with MBF and MFR values returning to normal only after up to around 21 and 20 min, respectively.

These findings contradict the common sense that MBF returns to normal within a few minutes after the termination of adenosine infusion. However, the clinical evidence supporting the common assumptions on the duration of adenosine-induced myocardial hyperaemia is, in fact, surprisingly scarce, with only a few historical studies addressing the effects of systemic adenosine application. All these studies measured coronary blood flow velocity using intracoronary Doppler catheters and demonstrated normalization within several minutes after adenosine, although with a relatively wide range of mean time to normalization ranging from 45 s in the study of Kern *et al*.,^8^ over 154 s as shown by Rossen *et al*.,^7^ to 310 s as documented by Wilson *et al*.^6^ Coronary blood flow velocity, however, does not reflect blood flow of the myocardium down to the level of arterioles but is instead a surrogate marker confined to (one or more) large coronary arteries. In contrast, quantitative PET MPI, as used in the present study, is considered the non-invasive gold standard for MBF measurements, allowing for an assessment of the entirety of the myocardial vasculature. This is corroborated by smaller studies performing head-to-head comparisons between coronary flow velocity reserve measurements and MFR as obtained from PET, whereby the overall correlations and agreement were only modest.^14,15^ Inarguably, the relationship between coronary blood flow velocity and MBF is complex and may be influenced by various factors such as vascular resistance, vessel diameter, and microvascular function, thus potentially explaining some of the differences to these previous studies.

Nevertheless, our findings may also seem surprising in light of the short elimination half-life of adenosine in the range of around 10 s, as it is rapidly deaminated or re-phosphorylated *in vivo*.^16,17^ However, adenosine does not exert its vasodilator capacity directly but rather through a complex adenosine receptor signalling cascade. Activation of G-proteins leads to stimulation of adenylyl cyclase, an increase of cyclic adenosine monophosphate (cAMP), and activation of protein kinase. The latter triggers adenosine triphosphate (ATP)-sensitive potassium (K_ATP_) channels, which hyperpolarize the smooth muscle cells, causing relaxation and, thus, vasodilation. Smooth muscle relaxation is further caused through increased cAMP by inhibiting myosin light chain kinase, which leads to decreased myosin phosphorylation and a decrease in contractile force. Finally, there is evidence that adenosine inhibits calcium entry into smooth muscle cells, leading to relaxation because of the reduction in intracellular calcium.^18,19^ Although it is beyond the scope of the present study to provide more insights into the physiology of adenosine, it is, in light of the said complex interplay of different mechanisms and second messengers, all contributing to coronary vasodilation while only being triggered by adenosine, not unlikely that the duration of the physiological reaction to adenosine, namely prolonged myocardial hyperaemia, is of much longer duration than the half-life of adenosine itself. Another potential mechanism promoted through prolonged regulation of the cardiovascular system cannot be entirely excluded by our data but seems unlikely, given that we did not find any correlation between the time interval between offset of adenosine and heart rate, systolic blood pressure, or the rate–pressure product.

In the domain of nuclear cardiology, and particularly with PET MPI, a rest–stress approach is most commonly applied in clinical routine. However, depending on institutional infrastructure, implementation of stress–rest protocols as an adjunct to rest–stress protocols may seem appealing because this approach yields distinct advantages in terms of logistics if, for example, scanning is performed on multiple PET scanners with some patients undergoing a stress–rest and some a rest–stress protocol in parallel, thus reducing the need for personnel while minimizing the per-patient cyclotron-incurred costs. However, this approach may come at the risk of a systematic artificial underestimation of MFR with potentially negative implications for our patients. Thus, the suggestion of current practice guidelines on PET MPI to refrain from rapid stress–rest protocols is supported by our results and should potentially be turned into a recommendation. If it is considered beneficial for a PET centre to implement both rest–stress and stress–rest protocols, this rationale should not be aimed towards time-saving but based on logistical considerations, as our results suggest that a sufficient time interval of at least 20 min should be maintained between adenosine termination and rest imaging in patients undergoing a stress-first protocol.

Our findings may hold important clinical relevance also outside the domain of nuclear cardiology because they pertain to all imaging modalities relying on adenosine infusion for MBF quantification. This is particularly true for stress CMR, where quantification of MBF is a rapidly emerging technique.^20,21^ Contrary to PET MPI, however, for CMR, the stress–rest approach is favoured for patient comfort and logistical reasons but also because it eliminates the possibility that stress images are contaminated by delayed enhancement from residual gadolinium-based contrast. In fact, a stress–rest protocol with time intervals as short as 5–10 min between acquisitions is regularly found in contemporary literature addressing quantitative myocardial perfusion CMR.^20–26^ According to our findings, the currently mandated waiting time of 10 min between stress and rest imaging^11^ may be too short to avoid residual effects from adenosine on rMBF measurements even if correction for residual contrast agent could be implemented.

## Conclusion

Intravenously applied adenosine induces a long-lasting hyperaemic effect on the myocardium, with a duration potentially lasting up to 20 min in some patients. Therefore, rapid stress–rest protocols should be avoided for MBF quantitation as rMBF may be overestimated and, consequently, MFR underestimated.

### Limitations

This is a retrospective single-centre study and, hence, exhibits all the potential limitations associated with this study design.

## Supplementary data

Supplementary data are available at *European Heart Journal - Cardiovascular Imaging* online.

## Supplementary Material

jeae096_Supplementary_Data

## Data Availability

The data underlying this article will be shared on reasonable request to the corresponding author.
