# Supplementary material for: Duration of adenosine-induced myocardial hyperaemia: insights from quantitative 13N-ammonia positron emission tomography myocardial perfusion imaging
Source: Eur Heart J Cardiovasc Imaging. 2024 Apr 8;25(10):1367–73. doi: 10.1093/ehjci/jeae096 (PMC11441031; doi:10.1093/ehjci/jeae096)
Supplement: jeae096_Supplementary_Data [file jeae096_supplementary_data.docx]

**TABLES**

**Table S1. MBF parameters and MFR without correction for the rate-pressure product**

|  |  | Imaging sequence | |  |
| --- | --- | --- | --- | --- |
|  | All patients (n=331) | Stress-rest (n=146) | Rest-stress (n=185) | p-value |
| rMBF_uncorrected_ (ml ∙ min^-1^ ∙ g^-1^) | 0.79 [0.65-1.01] | 0.86 [0.71-1.13] | 0.75 [0.62-0.93] | **< 0.001** |
| MFR_uncorrected_ | 3.07 [2.39-3.71] | 2.97 [2.29-3.54] | 3.19 [2.54-3.94] | **0.006** |

Values given are median and IQR in brackets.
